# Association of Neovascular Glaucoma with Risk of Stroke: A Population-Based Cohort Study

**DOI:** 10.1155/2017/1851568

**Published:** 2017-08-08

**Authors:** Cheng-Wen Su, Yue-Cune Chang, Cheng-Li Lin, Hsin-Yi Chen

**Affiliations:** ^1^Department of Ophthalmology, China Medical University Hospital, Taichung, Taiwan; ^2^Department of Mathematics, Tamkang University, Taipei, Taiwan; ^3^Management Office for Health Data, China Medical University Hospital, Taichung, Taiwan; ^4^School of Medicine, College of Medicine, China Medical University, Taichung, Taiwan

## Abstract

Neovascular glaucoma (NVG), caused by ocular ischemia, is a serious ocular disease complicated by intractably increased intraocular pressure. Cerebrovascular accidents are classified into ischemic and hemorrhagic stroke. Based on the similar pathogenic mechanisms of NVG and ischemic stroke, we investigated the relationship between NVG and stroke by using a nationally representative sample. This study included 416 NVG patients and 4160 controls. Medical comorbidities were also evaluated. The cumulative incidence of ischemic stroke was 15.6% higher in the NVG cohort than in the control cohort (*p* < 0.001); the incidence density rates of stroke were 3.80 and 1.19 per 10,000 person-years in the NVG and control cohorts, respectively. According to the multivariable Cox regression results, the estimated adjusted hazard ratio (aHR) of stroke was 2.07 (95% confidence interval (CI) = 1.41–3.02) for the NVG cohort. Furthermore, the NVG cohort was 2.24-fold more likely to develop ischemic stroke (95% CI = 1.51–3.32). The risk of ischemic stroke was higher in patients with hypertension (aHR = 2.09, 95% CI = 1.55–2.82) and in patients with diabetic retinopathy (aHR = 1.69, 95% CI = 1.05–2.72). Notably, patients with NVG have a higher risk of ischemic stroke, but not hemorrhagic stroke.

## 1. Introduction

Neovascular glaucoma (NVG), caused by ocular ischemia, is a serious ocular disease complicated by intractably increased intraocular pressure (IIOP). Ocular ischemia results from retinal vein occlusion, diabetic retinopathy (DMRP), or inflammatory ocular diseases. Additionally, the vascular endothelial growth factor (VEGF) level may increase in the anterior segment of the eye, leading to the neovascularization of the iris, angle, and trabecular meshwork [1–4] and thus inducing IIOP.

Cerebrovascular accidents, which are a global burden among aging populations [5], are classified into ischemic and hemorrhagic stroke. Ischemic stroke is caused by thromboembolic events, whereas hemorrhagic stroke results from ruptured cerebral vessels [6]. However, the inflammatory process in the vessels plays an important role in the pathogenesis of ischemic stroke [7]. Several inflammatory biomarkers, such as C-reactive protein (CRP), total homocysteine (tHcy), tumor necrosis factor receptor 2 (TNFR2), and VEGF, have been found to be associated with an increased risk of ischemic stroke [7].

Few studies have investigated the relationship between NVG and stroke. However, the similar pathogenic mechanisms of NVG and ischemic stroke warrant an exploration of the potential relationship between the two diseases. In this retrospective study, we examined this relationship by using a nationally representative sample obtained from the Longitudinal Health Insurance Research Database of Taiwan.

## 2. Patients and Methods

### 2.1. Data Source

This retrospective cohort study analyzed secondary data obtained from the Longitudinal Health Insurance Research Database 2000 (LHID2000). LHID2000 contains the health claims data of 1,000,000 beneficiaries randomly sampled from the Registry of Beneficiaries of the National Health Insurance (NHI) program of Taiwan. The universal NHI program was established in March 1995 and provides health-care coverage to more than 99% of the 23.74 million residents of Taiwan [8]. To ensure patient privacy, LHID2000 uses decoded patient identification numbers that are scrambled to enable academic and research access. This study was approved by the Institutional Review Board of China Medical University (CMUH-104-REC2-115).

### 2.2. Sampled Participants

**Figure 1:**
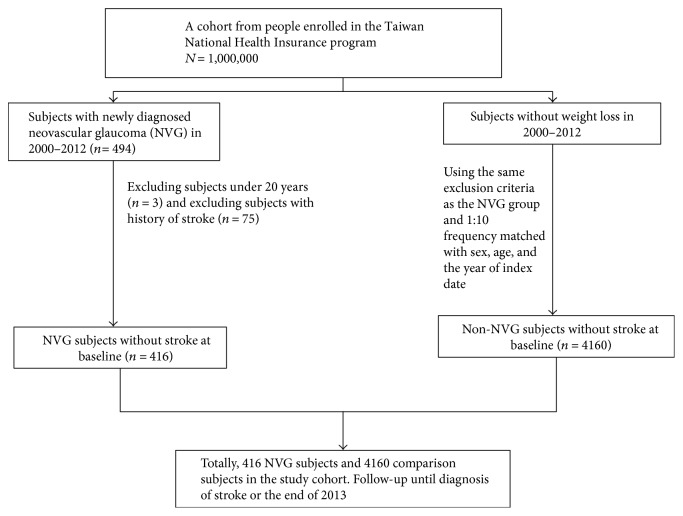
Schematic of selection process of participants in the NGV and control cohorts.

Figure 1 shows the selection process of participants in the NGV and control cohorts. From LHID2000, we identified patients aged 20 years or older who were newly diagnosed with NVG (International Classification of Diseases, Ninth Revision, Clinical Modification [ICD-9-CM] code 365.63) between January 1, 2000, and December 31, 2011. The date of NVG diagnosis was set as the index date. We excluded patients younger than 20 years, those with a history of stroke (ICD-9-CM codes 430–438) at baseline, and those without information on age and sex. Controls (randomly selected individuals without NVG) were frequency matched to patients with NVG at a 1 : 10 ratio by sex, age (5-year groups), and the year of the index date. The same exclusion criteria were applied to controls.

### 2.3. Outcome and Comorbidities

The endpoint of this study was the development of stroke (ICD-9-CM codes 430–438), which was subclassified into ischemic stroke (ICD-9-CM codes 433–438) and hemorrhagic stroke (ICD-9-CM codes 430–432) events. All patients were followed up from their index date to the endpoint, withdrawal from the NHI program, or until December 31, 2011. Baseline medical comorbidities included diabetes mellitus (DM) (ICD-9-CM code 250), hypertension (ICD-9-CM codes 401–405), hyperlipidemia (ICD-9-CM code 272), and coronary artery disease (ICD-9-CM codes 410–414). Baseline ocular comorbidities included central and branch retinal vein occlusion (ICD-9-CM codes 362.35 and 362.36), DMRP (ICD-9-CM code 362.0), retinal detachment with retinal defect (ICD-9-CM code 361.00), central and branch retinal artery occlusion (ICD-9-CM codes 362.31, 362.32, and 362.33), hypertensive retinopathy (ICD-9-CM code 362.11), and uveitis (ICD-9-CM codes 053.22, 054.44, 091.52, 364.0, 364.1, 364.2, 364.3, 360.0, 360.1, 091.5, 363.21, 360.04, 091.51, 094.83, 115.02, 115.12, 115.92, 130.2, 362.18, 363.0, 363.1, and 363.2).

### 2.4. Statistical Analyses

The chi-square test (or Fisher's exact test) and *t*-test were used to identify differences between the NVG and control cohorts for categorical and continuous variables, respectively. Cumulative incidence curves for ischemic stroke were plotted using the Kaplan-Meier method, and the log-rank test was used to identify the corresponding differences in curves between the NVG and control cohorts. We calculated the incidence density rates of stroke (per 10,000 person-years) for both cohorts, and the results were stratified by age, sex, and comorbidity. Univariable and multivariable Cox regression analyses were used to assess the hazard ratios (HRs), with 95% confidence interval (CI), of stroke for the NVG and control cohorts. Multiple models were used, mainly to adjust for the effects of sex, age, and comorbidities if they were determined to have a significant effect on the results (Table 1). All statistical analyses were conducted using SAS 9.4 software (SAS Institute, Cary, NC, USA), and all statistical tests were two sided; significance was set at *p* < 0.05.

## 3. Result

### 3.1. Demographic Characteristics and Comorbidities of the NVG and Control Cohorts

**Figure 2:**
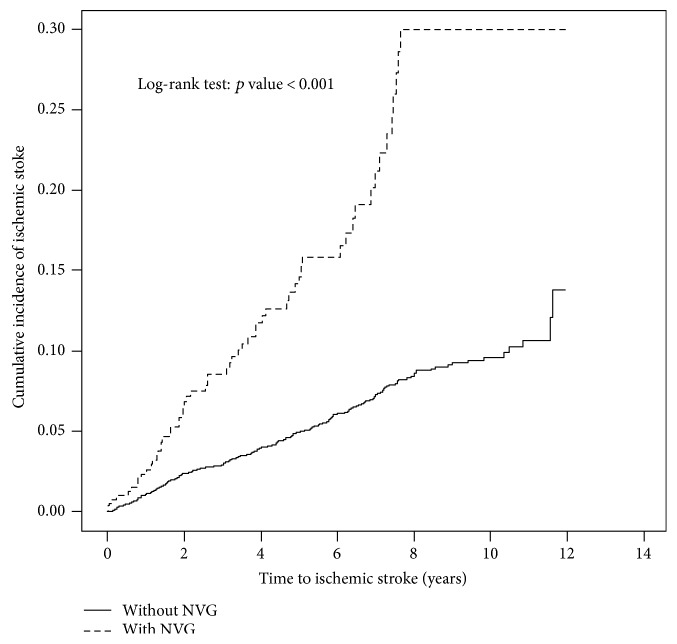
Comparison of the cumulative incidence of ischemic stroke between the NVG and control cohorts using the Kaplan-Meier method.

This study included 416 patients with NVG and 4160 controls who were recruited between 2000 and 2011. Of the 416 patients with NVG, 43.3% were older than 65 years and 57.9% were men (Table 1). The mean ages of the NVG and control cohorts were 61.5 (±14.2) and 60.9 (±14.6) years, respectively. Compared with controls, patients with NVG had a higher prevalence of medical and ocular comorbidities. The mean follow-up periods were 4.17 and 5.21 years for the NVG and control cohorts, respectively. As shown in Figure 2, the cumulative incidence of ischemic stroke was compared between the NVG and control cohorts by using the Kaplan-Meier method. By the end of follow-up, the cumulative incidence of ischemic stroke was determined to be 15.6% higher in the NVG cohort than in the control cohort (*p* < 0.001).

### 3.2. Incidence and HRs of Stroke Stratified by Age, Sex, and Comorbidity: Comparison between the NVG and Control Cohorts

The incidence density rates of stroke were 3.80 and 1.19 per 10,000 person-years in the NVG and control cohorts, respectively (Table 2). Compared with the control cohort, the multivariable Cox regression method's estimated adjusted HR (aHR) of stroke was 2.07 (95% CI = 1.41–3.02) for the NVG cohort. Furthermore, patients with NVG were 2.24-fold (95% CI = 1.51–3.32) more likely to develop ischemic stroke than controls after adjusting for the effects of other factors presented in the model. Similarly, compared with the control cohort, the aHR of stroke was 3.42- and 1.83-fold significantly higher for patients aged 50–64 years and for those older than 65 years in the NVG cohort, respectively (*p* < 0.001 and *p* < 0.01, resp.). In the sex-stratified analysis, the aHR of stroke for the NVG cohort versus the control cohort was 2.74 (95% CI = 1.63–4.61) for female patients (*p* < 0.001). Moreover, compared with those in the control cohort, patients in the NVG cohort with any one medical comorbidity had a significantly higher risk of stroke (aHR = 2.14, 95% CI = 1.43–3.19). Finally, according to the ocular comorbidity-stratified analysis, the risk of stroke was significant in patients without ocular comorbidities (aHR = 1.82, 95% CI = 1.10–3.03).

### 3.3. Univariable and Multivariable Cox Model Analyses of the Risk Factors for Ischemic Stroke

The results of univariable and multivariable Cox regression models for analyzing the risk factors for ischemic stroke are shown in Table 3. The aHR of ischemic stroke for the NVG cohort versus the control cohort was 2.24 (95% CI = 1.51–3.32, *p* < 0.001) after adjusting for the effect of other factors. Similarly, the aHR of ischemic stroke was significantly increased by 6% (95% CI = 1–1.06) for each increased year in age (*p* < 0.001). In the medical comorbidity-stratified analysis, the risk of ischemic stroke was significantly higher in patients with hypertension than in those without hypertension (aHR = 2.09, 95% CI = 1.55–2.82). By contrast, in the ocular comorbidity-stratified analysis, the risk of ischemic stroke was only borderline significantly higher in patients with DMRP (aHR = 1.69, 95% CI = 1.05–2.72; *p* = 0.032).

## 4. Discussion

Several studies have revealed that patients with glaucoma have higher risks of some systemic diseases. For example, primary open-angle glaucoma has been significantly associated with cerebral microinfarcts [9], stroke [10], and dementia [11]. However, few studies have investigated the relationship between NVG and other related systemic diseases. Our study is the first to show that the risk of stroke is significantly higher among patients with NVG than among people without NVG. Furthermore, our study revealed that compared with those without NVG, patients with NVG were 2.24-fold more likely to develop ischemic stroke, but not hemorrhagic stroke.

This finding is particularly important from a pathogenic perspective. The major etiology of NVG includes DMRP, central retinal vein occlusion, and ocular ischemic syndrome [4]. All of these conditions induce retinal ischemia, which produces the angiogenic factor of VEGF, and thus subsequently result in the growth of abnormal blood vessels in the iris and angle neovascularization [12]. The etiology of ischemic stroke is multifactorial, and major risk factors such as DM, HTN, smoking, and dyslipidemia play critical roles in stroke development [6]. These causative factors predispose patients to damage to their vascular endothelial cells, leading to the production of reactive oxygen species and inflammation; these processes also mediate thromboembolic events [6]. Shoamanesh et al. [7] found that the serum levels of four inflammatory biomarkers, namely, CRP, tHcy, TNFR2, and VEGF, are increased during incidents of ischemic stroke. In other words, from molecular and clinical perspectives, NVG and ischemic stroke have a similar pathogenesis. Moreover, VEGF may be a confounding factor. By contrast, hemorrhagic stroke results from the rupture of blood vessels, leading to leakage of blood in the brain; the rupture of blood vessels might result from several conditions affecting the blood vessels, including very high blood pressure (hypertension), overuse of anticoagulants, and weak spots in the blood vessel walls (aneurysms) [13].

The effect that sex has on the risk of stroke should also be addressed. In our study population, the aHR of stroke for the NVG cohort versus the control cohort was 2.74 (95% CI = 1.63–4.61) for women and 1.66 (95% CI = 0.95–2.89) for men. According to our review of the literature, stroke event rates are lower in women than in men, but sex comparisons based on age-adjusted rates mask key differences [14, 15]. In one study, researchers identified a higher lifetime risk of stroke among women than among men and noted a greater number of stroke deaths among women than among men [15]. Several woman-specific risk factors, including pregnancy [16], migraines, menopause, use of oral contraceptives, and hormonal replacement therapy [15–18], have also been determined to be related to stroke. Future prospective observational studies should focus on risk profile evaluation for establishing prevention strategies for women [15].

In contradiction to the presumed belief that aging is positively associated with the risk of stroke [5], our results showed that compared with those in the control cohort, the risk of stroke was significantly 3.42- and 1.83-fold higher among patients in the NVG cohort who were aged 50–64 years and among those older than 65 years, respectively. Usually, vision loss and intractable pain caused by IIOP prompt individuals with NVG to seek help from ophthalmologists. Thus, clinicians should be aware of the higher risk of stroke in younger individuals with NVG. Early neurological consultation is mandatory for preventing the onset of stroke.

Additionally, our results showed that patients in the NVG cohort with any one medical comorbidity had a significantly higher risk of stroke (aHR = 2.14, 95% CI = 1.43–3.19) than those with no comorbidities. However, further analysis indicated that among all medical comorbidities, the risk of ischemic stroke was significantly higher only in patients with hypertension (aHR = 2.09, 95% CI = 1.55–2.82). Evidence has shown that metabolic syndrome and its main definitional components, including high blood pressure and hyperglycemia, are strongly associated with an increased risk of stroke [19, 20]. Therefore, early identification and appropriate control of medical comorbidities are key strategies for preventing stroke in patients with NVG.

Our results also indicated that the risk of stroke was statistically significant in patients with NVG who did not have ocular comorbidities (aHR = 1.82, 95% CI = 1.10–3.03) and was significant in patients with NVG who had ocular comorbidities (aHR = 1.93, 95% CI = 1.00–3.75). However, further analysis indicated that the risk of ischemic stroke was only borderline significantly higher (*p* = 0.032) in patients with DMRP (aHR = 1.69, 95% CI = 1.05–2.72). The association between NVG and DMRP has been established clinically [4], and it is reasonable to state that NVG is an advanced manifestation of DMRP [4]. Treatment of DMRP with anti-VEGF agents has improved clinical outcomes [21, 22], and VEGF has been suggested to be a common circulating biomarker of NVG and ischemic stroke [7]. Further research is warranted to clarify the role that VEGF could play as a potential therapeutic target for preventing ischemic stroke.

Despite these promising results, our study has some limitations. First, LHID2000 does not contain detailed information on potential confounding factors, such as diet, smoking, obesity, lifestyle factors, or family history of glaucoma or stroke [14]; only common medical or ocular comorbidities could be included in our analyses. Second, although the sample size was large, the study cohort only comprised Taiwanese patients. Therefore, these findings cannot be easily applied to other ethnic groups [11]. Nevertheless, our study has some notable strengths. First, LHID2000 is an excellent source of data because of the large sample randomization, which enabled us to follow up with patients over time to assess the relationship between glaucoma and the subsequent onset of stroke [11]. Second, the database contains the data of people with diverse sociodemographic profiles, unlike previous smaller studies that recruited patients from specific regions and thus lacked representativeness [11]. Third, the possibility of misclassification was low in our study because NVG and stroke are both major medical illnesses. We believe that patients with both diseases would seek medical help.

In conclusion, patients with NVG have a higher risk of ischemic stroke, but not hemorrhagic stroke. Ophthalmologists should be aware of this risk when managing cases of NVG.

## Figures and Tables

**Table 1:** Comparison of demographics and comorbidities between patients with NVG and controls.

	NVG (*N* = 416)		Control (*N* = 4160)		*p* value
	*n*	(%)	*n*	(%)	
Age, year					0.99
≤49	87	(20.9)	870	(20.9)	
50–64	149	(35.8)	1490	(35.8)	
≥65	180	(43.3)	1800	(43.3)	
Mean (SD)^#^	61.5	(14.2)	60.9	(14.6)	0.36
Sex					0.99
Female	175	(42.1)	1750	(42.1)	
Male	241	(57.9)	2410	(57.9)	
Medical comorbidity					
Diabetes mellitus	224	(53.9)	531	(12.8)	<0.001
Hypertension	300	(72.1)	1681	(40.4)	<0.001
Hyperlipidemia	197	(47.4)	995	(23.9)	<0.001
Coronary artery disease	150	(36.1)	795	(19.1)	<0.001
Ocular comorbidity					
CRVO & BRVO	56	(13.5)	5	(0.12)	<0.001
DMRP	208	(50.0)	75	(1.80)	<0.001
Retinal detachment with retinal defect^§^	18	(4.33)	3	(0.07)	<0.001
CRAO & BRAO	5	(1.20)	0	0.00	—
HTR^§^	7	(1.68)	1	(0.02)	<0.001
Uveitis	66	(15.9)	25	(0.60)	<0.001

Chi-square test; ^#^*t*-test; ^§^Fisher's exact test.

**Table 2:** Comparison of the incidence density rates of stroke between patients with NVG and controls stratified by demographic characteristics and comorbidities.

	NVG						Crude HR (95% CI)	Adjusted HR^†^ (95% CI)
	Yes			No				
	Event	PY	Rate^#^	Event	PY	Rate^#^		
All	66	1736	3.80	257	21,658	1.19	3.21 (2.45–4.21)^∗∗∗^	2.07 (1.41–3.02)^∗∗∗^
Hemorrhagic stroke	8		0.46	30		0.14	3.24 (1.48–7.08)^∗∗^	1.15 (0.35–3.85)
Ischemic stroke	58		3.34	227		1.05	3.20 (2.40–4.28)^∗∗∗^	2.24 (1.51–3.32)^∗∗∗^
Age								
≤49	7	470	1.49	9	5561	0.16	9.91 (3.68–26.7)^∗∗∗^	1.51 (0.22–10.5)
50–64	25	583	4.28	57	7833	0.73	5.71 (3.56–9.17)^∗∗∗^	3.42 (1.97–5.95)^∗∗∗^
≥65	34	682	4.98	191	8264	2.31	2.18 (1.51–3.14)^∗∗∗^	1.83 (1.22–2.74)^∗∗^
Sex								
Female	35	735	4.76	103	9763	1.06	4.49 (3.05–6.60)^∗∗∗^	2.74 (1.63–4.61)^∗∗∗^
Male	31	1000	3.10	154	11,895	1.29	2.40 (1.63–3.53)^∗∗∗^	1.66 (0.95–2.89)
Medical comorbidity^&^								
No	3	367	0.82	55	11,476	0.48	1.67 (0.52–5.35)	1.20 (0.27–5.44)
Yes	63	1368	4.60	202	10,182	1.98	2.34 (1.76–3.11)^∗∗∗^	2.14 (1.43–3.19)^∗∗∗^
Ocular comorbidity^§^								
No	16	606	2.64	245	21,270	1.15	2.29 (1.38–3.80)^∗∗^	1.82 (1.10–3.03)^∗^
Yes	50	1130	4.42	12	388	3.09	1.43 (0.76–2.69)	1.93 (1.00–3.75)

^#^Incidence rate per 10,000 person-years; crude HR represents relative hazard ratio. ^†^Variables that were found to be significant in the univariable analysis and which were included in the multivariable analysis. ^&^Medical comorbidity (having at least one comorbidity classified as a medical comorbidity (e.g., diabetes mellitus, hypertension, hyperlipidemia, or coronary artery disease)). ^§^Ocular comorbidity (having at least one comorbidity classified as an ocular comorbidity (e.g., central and branch retinal vein occlusion, diabetic retinopathy, retinal detachment with retinal defect, central and branch retinal artery occlusion, hypertensive retinopathy, and uveitis)). ^∗^*p* < 0.05, ^∗∗^*p* < 0.01, ^∗∗∗^*p* < 0.001.

**Table 3:** Hazard ratios of ischemic stroke stratified by age and comorbidities in the univariable and multivariable Cox regression models.

Variable	Crude		Adjusted^†^	
	HR	(95% CI)	HR	(95% CI)
NVG	3.20	(2.40–4.28)^∗∗∗^	2.24	(1.51–3.33)^∗∗∗^
Age, years	1.07	(1.06–1.08)^∗∗∗^	1.06	(1.05–1.07)^∗∗∗^
Sex (female versus male)	1.08	(0.85–1.36)	—	—
Medical comorbidity				
Diabetes mellitus (yes versus no)	2.41	(1.86–3.12)^∗∗∗^	1.09	(0.78–1.53)
Hypertension (yes versus no)	4.27	(3.29–5.56)^∗∗∗^	2.09	(1.55–2.82)^∗∗∗^
Hyperlipidemia (yes versus no)	1.75	(1.37–2.24)^∗∗∗^	0.92	(0.70–1.22)
Coronary artery disease (yes versus no)	2.75	(2.16–3.50)^∗∗∗^	1.17	(0.90–1.52)
Ocular comorbidity				
CRVO & BRVO (yes versus no)	2.42	(1.08–5.45)^∗^	0.64	(0.27–1.51)
DMRP (yes versus no)	3.58	(2.58–4.98)^∗∗∗^	1.69	(1.05–2.72)^∗^
Retinal detachment with retinal defect (yes versus no)	1.04	(0.15–7.41)	—	—
CRAO & BRAO (yes versus no)	—	—	—	—
HTR (yes versus no)	2.78	(0.39–19.8)	—	—
Uveitis (yes versus no)	2.20	(1.17–4.14)^∗^	1.02	(0.53–1.99)

Crude HR represents the relative hazard ratio. ^†^Variables that were found to be significant in the univariable analysis and which were included in the multivariable analysis. ^∗^*p* < 0.05, ^∗∗∗^*p* < 0.001.
